# Nitrate of Pitocarpia

**Published:** 1878-02

**Authors:** 


Nitrate of Pilocarpia.—Physiological action of the infusion of jaborandi is so powerful and clearly defined that it is astonishing how little its therapeutic value has been turned to account. Nevertheless, it must be admitted that the study of this energetic agent presents serious difficulties, from the fact that its leaves are often adulterated, besides being frequently ill preserved, as found in the markets.

The discovery and isolation of pilocarpia, the active principle of the plant, will enable us, however, to arrive at the therapeutic indication for this drug; furthermore, the patient will no longer be nauseated by the disagreeable taste of the infusion, nor can the different results be attributed to a difference in the quality of the medicine.

In the present article we will confine ourselves to the physiological effects produced by the absorption of the alkaloid, and later refer to the conditions in which the practitioner should avail himself of this most powerful modifier of the salivary and cutaneous secretions.

Our experiments were made at the Hotel Dieu de Caen; we used the nitrate of pilocarpia hypodermically, the dose being from two to two and a half centigrammes (3-10 to $\frac{3}{8}$ grains). This salt is very soluble, and is therefore readily adapted to this form of medication.

The observations were made upon six patients, five of whom had some slight surgical trouble, and the sixth, a young man, suffered from phthisis pulmonalis. The following are the phenomena which we observed: one or two minutes, at most, after the injection, the patient felt flushes of heat in the face and a smarting sensation in the lumbar region; his countenance colored and his eyes were injected; a moment later, an abnormal secretion of saliva filled his mouth and caused him to spit for eight or ten seconds. The saliva is clear, watery and usually tasteless; sometimes, however, it is mixed with an abundant expectoration.

At the end of three minutes, small drops of sweat appeared upon the alæ of the nose; the brow presented nearly the same phenomenon; finally diaphoresis became general and sufficient to soil the mattress.

The thermometer placed in the axilla showed no elevation of temperature, 37° to 37½° (98 3-5° to 99° Fahrenheit). The pulse, on the contrary, was unsteady; in the space of ten minutes it rose to 140, only to fall in a quarter of an hour later to 75—generally it varied from 95 to 110.

In one case the heart palpitated forcibly, yet no murmur was heard.

Respiration was normal.

About ten minutes after the injection, three of the patients felt a desire to urinate; in each case the urine was diminished in amount, and the act of micturition caused a burning sensation in the urethral canal.

In every case vision was more or less disturbed, lachrymation was produced and sight was less distinct; objects appeared as though enveloped in a light veil.

As respects hearing, there was a sensation of buzzing and ringing in the ears, with beginning deafness. The digestive apparatus, with its attachments, also showed signs of disturbance; the tongue was slightly swollen and become painful at its base; there were nausea and vomiting, yet the latter was not abundant.

Thirty-five minutes after the appearance of the first phenomenon, the countenance was less flushed, the skin became cool, the extremities were cold, the limbs itched and the pulse was weak.

The patients were scarcely able to walk; they staggered and would have fallen, had they not supported themselves. The most feeble were completely exhausted, and, showing signs of stupor, they rested with their heads inclined over a basin receiving the saliva which, without effort, flowed from their mouths in abundance. Such are the curious effects which appeared in the first forty-five minutes, then little by little the secretion of saliva decreased, the perspiration become less copious, there was less feeling of malaise, and finally, at the end of an hour or two, everything had ceased.

The mean quantity of the saliva secreted by the patient was 500 grammes, (16 ounces) but in one case it even amounted to 900 grammes; the perspiation sufficient to saturate both mattress and covers.

The following fact is also rather peculiar: a woman received an injection of 0.025 milligramme (2-5 gr. nearly) of nitrate of pilocarpia in the arm; the above symptoms all appeared and passed away, but in the evening she had a second attack of sweating and salivation as abundant as the first. A soldier presented this same peculiarity, yet more strikingly, for in his case did the attack not only reappear in the evening, but also on the following morning, at an hour corresponding with the time of the injection on the day previous.

Such, then, are the physiological phenomena which we witnessed, and one can readily understand from the rapidity and certainty of action of this alkaloid, what position it will command in future therapeutics.—*L'Annee Medicale.*

